# An Update on the Potential of Tangeretin in the Management of Neuroinflammation-Mediated Neurodegenerative Disorders

**DOI:** 10.3390/life14040504

**Published:** 2024-04-14

**Authors:** Irshad Wani, Sushruta Koppula, Aayushi Balda, Dithu Thekkekkara, Ankush Jamadagni, Prathamesh Walse, Santhepete Nanjundaiah Manjula, Spandana Rajendra Kopalli

**Affiliations:** 1 Department of Pharmacology, JSS College of Pharmacy, JSS Academy of Higher Education & Research, Mysuru 570015, India; 2 College of Biomedical and Health Science, Konkuk University, Chungju-si 380-701, Republic of Korea; koppula@kku.ac.kr; 3 Fortem Biosciences Private Limited (Ayurvibes), No. 24, Attur, 4th Cross, Tirumala Nagar, A Block, Bangalore 560064, India; 4 Department of Integrated Bioscience and Biotechnology, Sejong University, Gwangjin-gu, Seoul 05006, Republic of Korea

**Keywords:** *Citrus* fruits, flavonoids, tangeretin, neuroinflammation, neurodegeneration, microglia, anti-inflammatory, antioxidant

## Abstract

Neuroinflammation is the major cause of neurodegenerative disorders such as Alzheimer’s and Parkinson’s disease. Currently available drugs present relatively low efficacy and are not capable of modifying the course of the disease or delaying its progression. Identifying well-tolerated and brain-penetrant agents of plant origin could fulfil the pressing need for novel treatment techniques for neuroinflammation. Attention has been drawn to a large family of flavonoids in citrus fruits, which may function as strong nutraceuticals in slowing down the development and progression of neuroinflammation. This review is aimed at elucidating and summarizing the effects of the flavonoid tangeretin (TAN) in the management of neuroinflammation-mediated neurodegenerative disorders. A literature survey was performed using various resources, including ScienceDirect, PubMed, Google Scholar, Springer, and Web of Science. The data revealed that TAN exhibited immense neuroprotective effects in addition to its anti-oxidant, anti-diabetic, and peroxisome proliferator-activated receptor-γ agonistic effects. The effects of TAN are mainly mediated through the inhibition of oxidative and inflammatory pathways via regulating multiple signaling pathways, including c-Jun N-terminal kinase, phosphoinositide 3-kinase, mitogen-activated protein kinase, nuclear factor erythroid-2-related factor 2, extracellular-signal-regulated kinase, and CRE-dependent transcription. In conclusion, the citrus flavonoid TAN has the potential to prevent neuronal death mediated by neuroinflammatory pathways and can be developed as an auxiliary therapeutic agent in the management of neurodegenerative disorders.

## 1. Introduction

The global economic impact of neurodegenerative illnesses is increasing considerably as life expectancy increases [1]. The pathogenic pathways that cause neurodegenerative diseases such as Alzheimer’s disease (AD), Parkinson’s disease (PD), and Amyotrophic lateral sclerosis (ALS) are not fully understood. Several factors are at work, including genetic, environmental, and endogenous impacts. Common pathogenic pathways include aberrant protein dynamics, oxidative stress with reactive oxygen species (ROS), mitochondrial dysfunction, DNA damage, neurotrophin dysfunction, and neuroinflammatory processes [2]. Neuroinflammation is a defense mechanism that initially protects the brain by removing or reducing different pathogens [3]. This inflammatory reaction may be advantageous, stimulating tissue repair and cleaning away cellular waste. Persistent inflammatory responses, on the other hand, are detrimental and hinder regeneration [4]. Inflammatory activation may persist due to endogenous (such as genetic mutations and protein aggregation) or exogenous (such as infection, trauma, and medicines) reasons [5]. Further, it is well known that microglia and astrocytes are engaged in persistent inflammatory responses that may lead to neurodegenerative diseases [6]. Despite extensive pre-clinical investigations, the outcome of stringent therapeutic strategies is poor.

Flavonoids, a group of natural chemicals with a polyphenolic structure, are present in almost 9000 varieties of plants. Familiar sources of these phytochemicals include fruits, tea, chocolate, vegetables, and many beverages [7,8]. Plant flavonoids are classified as flavanones, flavanols, isoflavonoids, flavones, chalcones, and anthocyanins according of their structural resemblance to two benzene rings joined by a heterocyclic ring and their availability as glycosides, aglycones, or methylated derivatives [9,10]. *Citrus* plants are the primary source of polymethoxyflavones (PMFs), flavones with two or more than 2 -OCH_3_ groups attached to the benzo-ˠ-pyrone (C_6_-C_3_-C_6_, 15-carbon ring) structure and in the C4th position, with one carbonyl group attached to the ring. Over 105 million metric tons of *Citrus* are produced annually around the world, with oranges making up more than half of that total.

Traditionally, *Citrus* peel, such as orange peel, is used by several countries in food, beverage, and cosmetic preparations, and to treat conditions like ringworm infections, stomach upsets, coughs, skin inflammation, changes in blood pressure, and muscle discomfort [11,12,13,14]. A prominent component, in addition to terpenoids and other volatile oils, is PMFs, which are abundant in *Citrus* peels [14]. *Citrus* flavonoids, especially methoxylated flavones [15,16], have shown immense therapeutic potential [17]. The most popular *Citrus*-plant-derived flavonoids include tangeretin (TAN), naringin, hesperidin, nobiletin, naringenin, and hesperetin. Among these, TAN, found only in *Citrus* fruit peels such as *C. aurantium* L., and *C. reticulata Blanco species*, is known to be used in traditional Chinese medicine, wherein TAN is the principal active component [18]. Ethnopharmacological studies on TAN have revealed numerous biological and functional properties, including antioxidant, anti-carcinogenic, anti-inflammatory, anti-atherogenic, and hepatoprotective properties, as well as actions on the neurological and proliferative fronts [19,20]. Pre-clinical studies have also indicated that TAN possesses neuroprotective and cognitive-enhancing benefits in cellular and in vivo experimental models including AD and PD [21,22,23,24]. Mechanistic-based studies indicated that TAN is involved in the regulation of multiple oxidative and inflammatory-related signaling pathways such as the inhibition of ROS, pro-inflammatory mediators, the regulation of NF-κB, MAPKs and JNK, and AKT activation [25]. These investigations indicated TAN’s potential role in regulating inflammatory-mediated signaling pathways.

Several studies have investigated the pharmacological importance and favorable biological activities of TAN in various disorders. Many reviews have explored the beneficial effects of TAN in terms of renoprotection, its anti-tumor, antidiabetic, immunomodulatory and antioxidant properties, and neuroprotection [7]. Further, the effects of TAN on the modulation of inflammation-mediated cancer-related pathways including PI3K/AKT/mTOR, Notch, and MAPK signaling, suppressing cell proliferation and inducing autophagy in various types of cancer cells, has also been addressed [10]. Although previous studies indicated the beneficial role of *Citrus*-fruit-derived flavonoids, including TAN, in neuroprotection [26], these studies mainly focused on the overall role of *Citrus* flavonoids in cholinergic regulation, regulating N-methyl-D-aspartate (NMDA) receptor hypofunction, ischemic injury, and oxidative stress signaling in toxin-induced models, with little emphasis on TAN and its pathways in neuroinflammation [26]. In this review, we aimed to understand the recent updates regarding the potential of TAN, its physiochemical characteristics, its safety profile, its pharmacokinetic parameters and the underlying molecular mechanisms involved in targeting neuroinflammation-mediated neurodegenerative disorders. The data for the review were collected using various literature resources, including ScienceDirect, PubMed, Google Scholar, Springer, and Web of Science.

## 2. Chemistry and Sources of TAN

**Figure 1:**
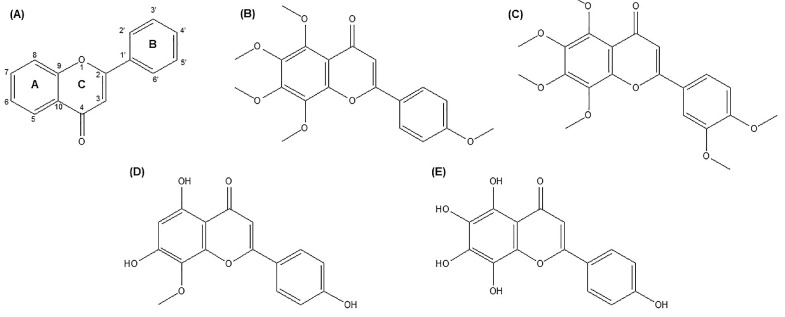
Citrus flavonoid structures. (**A**) Flavone skeleton, (**B**) tangeretin, (**C**) nobiletin, (**D**) apigenin, and (**E**) nortangeretin.

Natural O-methylated flavones, known as PMFs, primarily found in *Citrus* plants, are well-known for their health-improving qualities. In *Citrus* plants and other natural sources of PMFs, the enzyme O-methyltransferases is involved in manufacturing PMFs [27]. At least two methoxy groups are present on the flavone skeleton in PMFs (Figure 1A). Among the approximately 80 PMFs found in plants, TAN (Figure 1B) and nobiletin (Figure 1C) are the two most common [27]. TAN, a polymethoxy-flavonoid (5,6,7,8,4′-pentamethoxy flavone), is a low-molecular-weight (average mass 372.369 Daltons and molecular formula C_20_H_20_O_7_) secondary metabolite that is a member of the flavonoid class of polyphenols. The International Union of Pure and Applied Chemistry’s (IUPAC’s) name for TAN is 5,6,7,8-tetramethoxy-2-(4-methoxyphenyl)-chromen-4-one. Within the flavone structural subclasses, apigenin (Figure 1D) is TAN’s most straightforward structural counterpart. The presence of two aromatic rings (rings A and B), connected by a three-carbon chain that cyclizes to form ring C, a double bond at the C2/C3 position, and the absence of oxygenation at the C-3 position are the distinguishing structural characteristics of flavones. TAN is most likely produced synthetically by methylating all the hydroxyl groups of its nearest structural counterpart, i.e., Nortangeretin (5,6,7,8,4′-pentahydroxyflavone; Figure 1E) [26].

The polymethoxylated flavonoids, including TAN, are widely distributed in citrus fruits and notably rich in fruit peels. Numerous other, smaller-scale, natural sources of TAN have been noted, including Fructus species [28]. TAN and other related polymethoxylated flavonoids are frequently used for quality control of citrus juices due to their abundance in citrus fruits and variable concentrations between species. TAN is currently primarily consumed through the consumption of *Citrus* fruits. *C. tangerina*, *C. sinensis*, and *C. aurantium* fruit peels are highly known for their high levels of TAN, as well as the 4′-methoxylated derivatives of TAN and nobiletin. TAN, hesperidin, and beta-sitosterol have been extracted from *C. jambhiri* fruit peel, TAN, hesperidin, and beta-sitosterol [29]. The *Citrus* fruit bergamot, *C. bergamia*, also contains TAN [30]. TAN has also been found in several different types of *Citrus* species, including *C. unshiu*, *C. reticulata*, *C. tachibana*, *C. depressa*, *C. paradisi* (grapefruit) [31], and *C. poonensis* [32]. The sources of TAN are summarized in Table 1.

Many laboratories have concentrated on developing extraction procedures for TAN and related polymethoxylated derivatives due to the multiple pharmacological actions of TAN and related compounds that have been identified in the last few decades. These molecules have high lipophilicity, making it convenient to extract them using organic solvents and supercritical CO_2_ [33]. A comprehensive synthetic strategy has also been developed to create a consistent chemical supply of these compounds [34].

**Table 1:** Naturally occurring sources of TAN from plants (* study discovered PMFs but not explicitly TAN).

TAN Source	Plant Part	Reference
*Citrus poonensis* (*C. poonensis*)	Peel	[32]
*C. exocarpium Rubram*	Exocarp	[35]
*C. reticulata*	Peel	[36]
*C. unshiu*	Peel	[37]
*C. depressa Hayata*	Peel	[38]
*Hura crepitans*	Leaves, bark, roots	[39]
*Fructus aurantia*	Fruit	[28]
*C. aurantifolia*	Peel, fruit pulp	[40]
*C. mitis Blanco*	Peel	[41]
*C. aurantium*	Peel	[42]
*C. reticulate Cv. Suavissima*	Fruit	[43]
*C. grandis Osbeck*	Leaves	[44]
*C. reticulata* *, *Citrus paradisi*	Fruit	[45]
*C. clementina*		
*C. sinensis*		
*C. paradise*		
*C. lumia Risso*		
*C. ichangensis Swingle* *	Peel	[43]

### 2.1. Physicochemical Properties of TAN

TAN has a melting point of 154 °C, and a solid, light yellow, needle-like appearance with a 372 M + EI-MS *m*/*z* ratio [46]. TAN has hydrophilicity because of the polarity of its pyran ring. All the additional functional groups that extend from the benzene rings are CH_3_ groups, which give the cell membrane a high degree of permeability [7]. In bergamot oil, TAN is highly soluble, especially at 60 °C or higher. It is insoluble in water but soluble in methanol or ethyl acetate. The applications of TAN in pharmaceutical or food science are limited by its poor water solubility and unpleasant taste [47,48]. The various physicochemical properties of TAN are summarized in Table 2. The solubility and passive permeability of TAN were found to be 19 µg/mL (low soluble) and 1.62 × 10^−6^ cm/s (highly permeable) using LYSA and PAMPA methods [49,50].

### 2.2. Metabolism and Pharmacokinetic Profile of TAN

**Figure 2:**
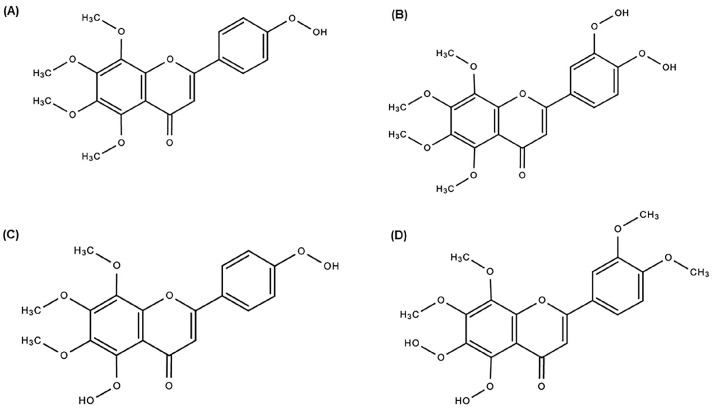
Metabolites of tangeretin: (**A**) 4′-dihydroxytangeretin, (**B**) 3′,4′-dihydroxylnobiletin, (**C**) 5,4′-dihydroxytangeretin, and (**D**) 5,6-dihydroxylnobiletin.

The biotransformation of PMFs in vivo results in the production of metabolites and has various pharmacological characteristics. The discovery and metabolism of these PMF metabolites has attracted attention. The primary enzyme system involved in the metabolism of PMFs is cytochrome P450 (CYP), which can catalyze the hydroxylation and demethylation processes. It is believed that all *Citrus* species have the same metabolic process for producing PMFs. The number and position of hydroxyl and methoxyl groups significantly affect how PMFs are metabolized [14]. In an in vitro experiment, when TAN was incubated with Aroclor-induced rat liver microsomes, the three primary metabolites of TAN were identified as 4′-dihydroxytangeretin (Figure 2A), 3′,4′-dihydroxylnobiletin (Figure 2B), and 5,4′-dihydroxytangeretin (Figure 2C) [51]. However, four of the five metabolites in a different in vitro metabolism study using human CYP were demethylated at position 4′, with 4′-demethyltangeretin being the principal metabolite. It was interesting to note that 5,6-dihydroxylnobiletin (Figure 2D) was the second most abundant metabolite [52].

Ten metabolites were found in an in vivo biotransformation investigation of TAN using repeated gavage feeding on rats. Seven of the ten identified metabolites, including the significant metabolites 4′-demethyltangeretin and 3′,4′-dihydroxylnobiletin, were demethylated at position 4′. The second most typical location for demethylation was position 6 of ring A. Therefore, it may be inferred that TAN position 4′ is the primary site for demethylation, while position 6 is the secondary site. Additionally, the investigation determined that glucuronate–sulfate conjugates make up 38% of the TAN metabolites that are eliminated in the urine [53].

Since TAN demonstrates significant absorption and is consequently bioavailable, it offers a particular advantage over other chemically identical flavones [54]. Furthermore, the oral administration of TAN is safe [55]. Hung et al. [56] found the plasma level of TAN to be 87 ± 0.33 μg/mL (C_max_) following oral treatment (50 mg/kg b.w.), with T_max_ and t_1/2_ values of 340 ± 48.99 and 342.43 ± 71.27 min, respectively. On the other hand, the plasma concentration of TAN after intravenous (5 mg/kg b.w.) administration was 1.11 ± 0.41 μg/mL, with T_max_ and t_1/2_ values of 1.11 ± 0.41 and 69.87 ± 15.72 min, respectively. After the oral and intravenous administration of TAN, the AUC values were 213.78 ± 80.63 and 78.85 ± 7.39 min μg/mL, and the absolute oral bioavailability was 27.11%. Additionally, only TAN concentrations of 0.0026 and 7.54% were found in the urine and feces after oral dosing, indicating that it was eliminated as a metabolite. The kidney had the highest concentration of TAN, followed by the lung, liver, spleen, and heart. Furthermore, the stomach and small intestine had the highest concentration at 4 h, while the cecum, colon, and rectum had the highest concentration at 12 h in the digestive tract [56].

In another investigation, when TAN was administered intravenously (10 mg/kg b.w.) to rats, the C_max_ was 2470 ± 557 ng/mL with a t_1/2_ value of 166 ± 42 min and was rapidly cleared. When administered as suspension (50 mg/kg b.w.), poor bioavailability was observed. However, an oral dose of 50 mg/kg body weight resulted in a C_max_ of 65.3 ± 20.1 ng/mL and a T_max_ value of 90–120 min. When TAN was prepared with methylated β-cyclodextrin, the oral bioavailability improved (C_max_: 135 ± 46 ng/mL) and the T_max_ value reduced to 30–90 min. This formulation increased the absolute oral bioavailability twofold (6.02%). Additionally, this research revealed that TAN was stable (recovery rates of 87–100%) under different storage settings [57]. Furthermore, this indicated that the plasma concentration of TAN (50 mg/kg b.w.) was the highest at 30 min, at 4.5 μg/mL, and declined to 0.65 μg/mL after 12 h following oral or i.p. administration [58].

### 2.3. Safety and Toxicity of TAN

TAN was used as a model compound for safety evaluations to examine the possibility of oral toxicity since it is one of the most common PMFs obtained from natural sources. To established TAN-induced acute oral toxicity in mice, doses of 1000, 2000, and 3000 mg/kg of TAN in an oil solution were administered to mice via gavage. Fourteen days following the injection, no fatalities were noted. However, daily, low-dose TAN administration led to a U-shaped dose–response curve with liver changes. Thus, PMF (a component of the human diet) may be employed safely under various circumstances [59].

In 2005, Vanhoecke et al. supported the results of earlier work by demonstrating the safety of TAN when administered orally to experimental mice. One piece of evidence was the absence of significant organ damage or a decline in function. These findings paved the way for subsequent human safety assessments [55]. In another study, the potential genotoxicity of TAN, determined by adding various concentration of PMF mixture to five distinct bacterial strains in vitro, was evaluated. According to the results, no mutations were found, regardless of whether ribosomal protein S9 was activated. The reported results demonstrated a good safety profile for the PMF combination with no chance of genotoxicity when using in vitro test methods [60]. However, the same group discovered a statistically insignificant positive connection between rising PMF concentrations and mice’s spleen weight in a separate investigation where mice were inoculated with sheep’s red blood cells (SRBCs). There was no proof that the spleen weight changed in mice without vaccination [61].

## 3. Neuroinflammation and Therapeutic Potential of TAN

Neuroinflammation-related neurodegenerative disorders such as PD and AD have become a significant burden as the population ages. In addition to being a pathogenic element causing many neurodegenerative illnesses, neuroinflammation is an essential biological response to neuronal tissue dysfunction [62]. The severity and frequency of neuroinflammation are the leading indicators of whether it will progress [63].

Microglia are the principal immune cells in the central nervous system (CNS), and under physiological conditions, these act similarly to macrophages. They serve as the initial line of immune defense for neurons, followed by tissue healing. But excessive microglial activation can result in neuronal death, a hallmark of neurodegenerative disorders, due to the ensuing robust cytokine production. Chronic or traumatic stresses, age-related microglial sensitization, and extended microglial activation are linked to neuroinflammation’s pathological and destructive course [62,63]. Excessive cytokine production, which leads to the elevation of various cytokine-regulated signaling systems involved in inflammation, apoptosis, and autophagy, underlies the disturbed communication balance between the brain and the immune system. One of these signaling systems is the nuclear factor-κB (NF-κB) pathway, whose deregulation has already been connected to the pathophysiology of PD and AD [64,65]. Further, it is well understood that the neurophysiological function of the CNS is critically dependent on astrocytes. Astrocytes are a double-edged sword in neurodegenerative disorders. When pathological alterations associated with neurodegenerative diseases increase, inflammatory responses stimulate astrocytes and convert them into an activated state [66,67]. The presence of reactive astrocytes was observed in AD and PD experimental models, wherein they released pro-inflammatory cytokines and neuroinflammatory mediators such as TNF-α, IFN-γ, IL-1, and IL-6, leading to harmful effects and directly contributing to neurodegeneration [67,68,69].

The mitogen-activated protein kinase (MAPK) family [70] and the phosphatidylinositol 3-kinase (PI3K)/serine/threonine kinase (Akt)/mammalian target of the rapamycin kinase (mTOR) signaling cascade are two additional pathways regulating inflammatory response and apoptosis, whose dysfunction is crucial in transforming neuroinflammation from a biological course to a pathological response [71]. ROS, nitric oxide (NO), and prostaglandins (PGs) are secondary messengers that activate these pathways and also act as proinflammatory mediators.

On the one hand, increased ROS production from hyperactivated microglia induces the release of cytokines [62]. On the other hand, cytokines promote the generation of ROS in the mitochondria [72], which can act as secondary messengers to activate the NF-κB and MAPK family pathways [73]. Oxidative stress may also lead to the expression of an inducible isozyme of nitric oxide synthases (iNOS), resulting in excess NO that damages cell proteins, ultimately leading to neuronal cell death [74]. Currently available and advised pharmacological treatments treat neuroinflammation as a concurrent pathology of neurodegenerative illnesses like AD. The cyclooxygenase (COX) pathway is also a key participant in neuroinflammation. It is interesting to note that the increased production of prostaglandin E2 (PGE2), a highly potent neuroinflammatory mediator, is mediated by both constitutive COX-1 and inducible COX-2 isoforms. The stimulation of stress-responsive pathways, such as the NF-κB pathway, is related to COX-1 upregulation and microglia activation, whereas the overexpression of COX-2 results in direct neuronal injury [62,75].

**Figure 3:**
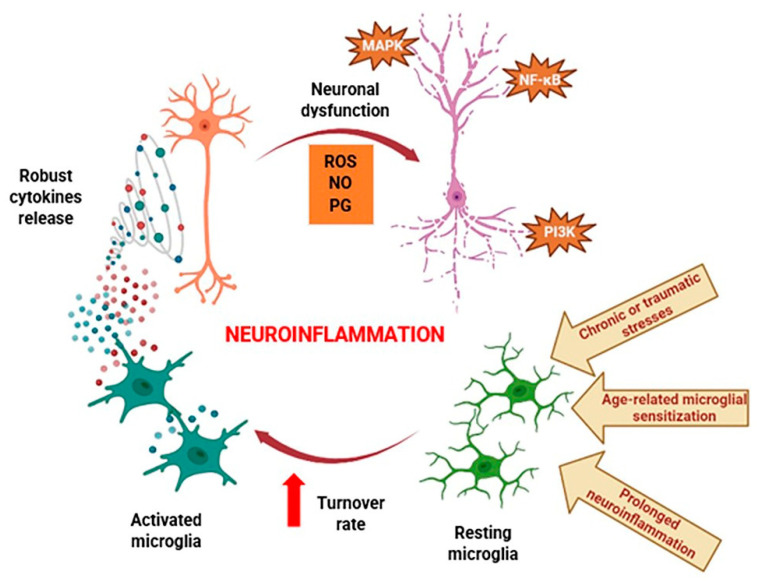
Schematic overview of pathologic neuroinflammation progression. Chronic or traumatic stresses, age-related microglial sensitivity, and the protracted inflammation of neurons, which cause the significant release of cytokines, significantly enhance the overall turnover rate of microglia. Cytokines and secondary messengers such as ROS, PG, and NO interfere with nerve cells’ ability to function normally. NF-κB, PI3K, and MAPK pathway failures are the first cause of progressive cell degeneration. ROS: reactive oxygen species; PG: prostaglandins; NO: nitric oxide; NF-κB: nuclear factor-kapa B; PI3K: phosphoinositide 3-kinase; MAPK: mitogen-activated protein kinase.

Given the various crosstalk across signaling cascades and the proinflammatory mediator’s interchangeable functions as an upstream activator and downstream effector in inflammatory signal transduction, it is clear that the process of neuroinflammation is exceptionally complex. The pathogenic development of neuroinflammation is schematically depicted in Figure 3.

Currently available treatments for neuroinflammation include traditional anti-inflammatory drugs [76,77,78,79], modulators of tumor necrosis factor (TNF-α)/NF-κB signaling [80,81,82,83], and plant-based natural therapies [84]. The urgent need for novel treatment approaches in neuroinflammation, exacerbated by the steadily aging global population, has led to the search for well-tolerated and brain-penetrant anti-inflammatory drugs of plant origin. Thus, scientists are interested in a vast family of flavonoids that are present in edible fruits because they have the potential to function as potent nutraceuticals that can delay the onset and progression of illnesses associated with neuroinflammation. There is mounting evidence that *Citrus* PMFs intervene in neurodegeneration and enhance brain functions, which are traits of neuroprotective agents.

PMFs, particularly TAN, have drawn the attention of natural product scientists due to their broad spectrum of bioactivities. Some of the bioactivities of PMFs include anti-inflammatory, anti-cancer, and anti-photoaging properties, the thermogenesis of white and brown adipose tissues, attenuation of the metabolic syndrome, skeletal muscle damage prevention, control of the gut flora, and neuroprotective and anti-atherogenic qualities [85,86,87,88,89,90,91,92,93].

### 3.1. Effect of TAN in PD Models

PD, which affects about 1% of people over 60 and over 10 million people globally, is a multisystem neurodegenerative disorder with the progressive loss of midbrain dopamine (DA) neurons and subsequent dopaminergic deafferentation of the basal ganglia, which results in recognizable motor disturbances like the slowing down of movement, rigidity of the muscles, and resting tremors [94,95]. In the following section, we outline the available literature and scientific studies on the potential of TAN against PD.

The first in vivo study to demonstrate that TAN can effectively cross the blood–brain barrier and has a neuroprotective effect on the brain was studied by Dexter et al. at the Department of Neuroinflammation, Imperial College of Science, London [21]. The authors reported that the chronic administration of TAN (10 mg/kg/day for 28 days) led to a marked increase in the levels of TAN in various brain regions of rats, including the hypothalamus, hippocampus and striatum. Further, TAN improved striatal dopamine depletion and reduced the 6-hydroxydopamine (6-OHDA)-induced decline in tyrosine hydroxylase-positive (TH^+^) cells.

A previous report on an experimental PD model showed that TAN (50, 100, or 200 mg/kg body weight) treatment for 20 days after MPTP intoxication was induced in rats significantly improved motor dysfunction, memory impairments, and cognitive disabilities. Further, the altered levels of proinflammatory cytokines in MPTP-induced rats were reversed by lowering the levels of inflammatory cytokines including interleukin (IL)-1β, IL-6, and IL-2. TAN treatment recovered the dopaminergic neuronal degeneration and hippocampus neuronal loss. The authors indicated that the positive effects of TAN might be achieved through inhibiting the TNF-α, COX-2, and iNOS signaling pathways in an MPTP-induced rat PD model [48].

In addition, after chronic 1-methyl-4-phenyl-1,2,3,6-tetrahydropyridine/probenecid (MPTP/P) injections in mice, TAN increased the messenger ribonucleic acid (mRNA) levels of unfolded protein response (UPR) target genes in dopaminergic neurons and astrocytes and enhanced neuronal protection [96,97]. The data were convincing and in agreement with previous studies suggesting that neuroprotective agents targeting neuroinflammatory pathways in PD pathology were known to preserve the striatonigral integrity partially mediated by activated astrocytes [98,99].

In a transgenic *Drosophila* PD model, abnormal activity patterns were observed in flies, including a reduced climbing ability, an increased concentration of dopamine, and disrupted antioxidative enzyme status, when compared with their respective controls. However, when TAN (5, 10, and 20 µM) was provided in the diet of PD model transgenic flies for twenty-four days, the authors found that the altered climbing ability and dopamine levels were better restored compared with the results obtained using similar doses of L-Dopa, which was used as a standard. Further, the altered antioxidative defense parameters were positively regulated with TAN treatment. These results indicate that TAN is indeed able to reduce the PD symptoms in experimental models and could be further clinically explored [100].

An increased risk of incident epileptic seizures has been reported in individuals with incident Parkinson’s, according to retrospective cohort research conducted in the United Kingdom. When compared to individuals without PD who do not have any seizure-provoking comorbidities, researchers discovered that PD patients with other brain disorders or multiple seizure-provoking comorbidities had the highest chance of epileptic seizures [101]. TAN treatment at 50, 100, or 200 mg/kg for 10 days in pilocarpine (30 mg/kg)-induced mice was shown to downregulate the suppression of PI3K/Akt signaling. The authors indicated that TAN alters neuronal apoptosis and ameliorates seizure severity in epilepsy-induced rats by enhancing the PI3K/Akt signaling pathway, reducing seizure-induced matrix metalloproteinases-2 (MMP-2) and matrix metalloproteinases-9 (MMP-9) activation, and lowering the level of apoptosis-inducing factor (AIF) in the nucleus via blocking AIF translocation [102]. These data indirectly suggest that TAN might be helpful in PD patients with epileptic seizure conditions.

### 3.2. Effect of TAN in AD Models

AD is a multifaceted, intricate CNS disorder that includes both neurodegeneration and persistent neuroinflammation, along with a significant loss of memory and cognitive functions. Although research on the beneficial effects of TAN in relation to AD and AD pathology are limited, some of the published data indicate that it can play a positive role in the management of AD pathology. In recent study by Shu et al. at the Institutes of Biomedical Sciences, School of Medicine, Jianghan University, China, the effects of TAN in an AD model of mice were evaluated [22]. The authors found that TAN treatment (100 mg/kg body weight/day) significantly ameliorated the cognitive dysfunction and reduced amyloid beta (Aβ) aggregation and synaptic loss in an APPswe/PSEN1dE9 transgenic (Tg) mice AD model. A mechanistic study revealed that TAN could inhibit β-secretase in both in vitro and in vivo studies, proving TAN’s potential role in the management of AD pathogenesis [22].

In a recent report, Prof. Hsu et al. [103], from the Department of Food Science, National Ilan University, Taiwan, evaluated the effect of TAN and nobiletin against Aβ-induced toxicity in primary rat neurons. TAN and/or nobiletin were used to treat primary cortical neuron toxicity induced by Aβ_1–42_. The authors indicated that TAN (25 µM) exhibited stronger neuroprotective effects when compared with nobiletin by attenuating the free radical damage induced by Aβ, thereby reducing intracellular oxidative damage. Additionally, TAN suppressed the Aβ_1–42_ monomer aggregation, indicating its potential as a neuroprotective agent [103].

### 3.3. Effect of TAN on Ischemic Brain Injury Models

It is commonly known that ischemic stroke is caused by the brain damage brought on through the blockage of the cerebral arteries after an extended period of ischemia. Further, reports have suggested that citrus flavonoids influence cardiovascular function and cerebral ischemia, making them especially pertinent in relation to neurodegenerative disorders [104,105]. In human hepatocellular carcinoma cells (HepG2) subjected to hypoxic conditions, TAN was shown to improve cell viability and decrease apoptosis. Further, Lee et al. [18] from Kyungpook National University, Daegu, Republic of Korea, studied the natural herb *Aurantii immatri* pericarpium, containing nobiletin and TAN as major constituents, for its brain-protecting effects in a rat model of ischemia-reperfusion. The authors indicated that TAN shielded the brain from damage by inhibiting apoptosis in human hepatocellular carcinoma cells (HepG2) and ameliorating brain injury in an ischemic-reperfusion model of rats [18].

Recently, Zhang et al., from the Department of Neurology, Huaihe Hospital of Henan University, Kaifeng, China, proved that TAN protected human brain microvascular endothelial cells (HBMECs) against oxygen–glucose deprivation (OGD) insult. TAN increased the superoxide dismutase activity and HBMEC survival while decreasing ROS and MDA levels. These effects were produced via suppression of the neuroinflammatory JNK signaling pathway [106].

In a much recent study, the effect of TAN on cognition and memory deficits in a cerebral ischemia rat model was studied [24]. The authors indicated that the decrease in cognitive and motor behavioral functions in bilateral common carotid artery occlusion (BCCAO) and reperfusion injured rats was ameliorated by TAN. Further, TAN improved the altered acetylcholine enzyme activity, oxidative enzyme status, inflammatory mediators, and apoptotic biomarkers in BCCAO rats. The authors concluded that TAN has potential neuroprotective effects against cerebral ischemia.

Further, considering the neuroprotective effects of TAN, Zan et al. [107], from the School of Biomedical and Pharmaceutical Sciences, Guangdong University of Technology, China, investigated the effects of TAN on cerebral ischemia reperfusion-induced neuronal injury in vivo in a middle cerebral artery occlusion/reperfusion (MCAO/R) mice model and oxygen–glucose deprivation and reoxygenation (OGD/R) injury in a hippocampal HT22 cell in vitro model. The authors claimed that TAN significantly attenuated cerebral ischemia and reperfusion (I/R) injury-induced neuronal absent in melanoma 2 (AIM2) inflammasome activation-mediated brain damage and inhibited pyroptosis in mice. Further, TAN regulated NRF2 signaling in hippocampal HT22 cells, indicating that TAN inhibits AIM2 inflammasome activation by the regulating NRF2 pathway. These studies provide strong insights into the therapeutic benefits of TAN in I/R-induced brain damage [107]. A summary of the established neuroprotective effects of TAN in experimental models of neuroinflammation and neurodegeneration is provided in Table 3.

### 3.4. Effect of TAN on Neurogenesis and Cognitive Functions

It is widely known that several neurodegenerative diseases dysregulate cAMP response element (CRE) transcription [109]. Additionally, the cAMP/CREB/ERK/PKA signaling pathway is vital for memory and learning [110]. In PC12D cells [111], and hippocampus neurons [112], TAN increases nerve outgrowth and induces CRE-dependent transcription. These data show that TAN may induce neuroprotection in neuronal cells through CRE-mediated transcription coupled with the upstream cAMP/CREB/ERK/PKA pathway.

In a previous study from Wu et al. at the Institute of Molecular Rhythm and Metabolism, Guangzhou University of Chinese Medicine, China, the authors explored the cognitive enhancing properties of TAN in a delirious mice model by injecting mice with an LPS plus midazolam (LM) model [23]. The impaired cognitive and attentional functions in delirium-induced mice were challenged by the administration of TAN at various doses (5, 10, and 15 mg/kg). Mice with LM-induced delirium exhibited decreased attentional and cognito-behavioral functions and TAN significantly ameliorated these changes. Mechanistic studies revealed that TAN functioned as an RORα/γ agonist, leading to enhanced memory and cognition in LM-delirium mice. Further, the increased expression of neuroinflammatory pathways, including ERK ½, TNF-α, and IL-1β, was reduced in LPS-stimulated microglia. The authors suggest that TAN regulates the expression of RORα/γ-related genes such as *E4bp4* and *Bmal1*, thereby promoting enhanced cognition in mice with LM-induced delirium, indicating the importance of TAN in preventing memory and cognitive deficits.

**Figure 4:**
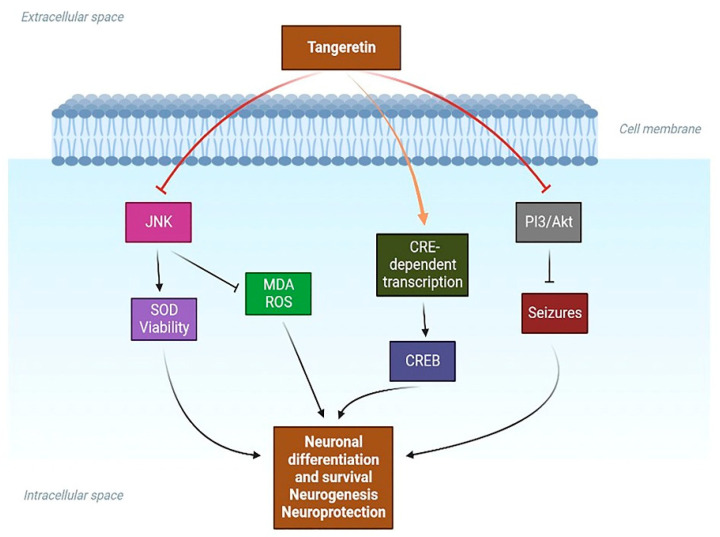
Signaling pathways involved in the neuroprotective benefits of TAN. PI3K: phosphoinositide 3-Kinase; SOD: superoxide dismutase; JNK: c-Jun N-terminal kinase; MDA: malondialdehyde; CREB: cAMP-response element binding protein; ROS: reactive oxygen species.

In a recent study, the effect of TAN on brain injury induced by chromium was evaluated [108]. Potassium dichromate, a xenobiotic, was administered to Wistar rats and the neuroprotective potential of TAN was explored. The altered inflammation and oxidative stress parameters were studied. Potassium-dichromate-induced changes in the brain’s MDA and GSH levels, as well as the increased levels of TNF-α and IL-6, were significantly reversed in TAN-treated subjects. Furthermore, this study revealed that TAN significantly reversed the potassium-dichromate-induced alterations in behavior and cholinergic activities. The authors suggest that the mechanism involved in the potential neuroprotective benefits exhibited by TAN might be the regulation of the Nrf2 and caspase-3 signaling pathways [108]. A schematic diagram of the possible signaling pathways involved in the neuroprotective benefits of TAN is shown in Figure 4.

## 4. Other Supporting Mechanisms of TAN in Neurodegeneration

### 4.1. Antioxidant Effect of TAN

Numerous diseases or pathological changes, such as cancer and metabolic problems, are linked to oxidative damage, including neurodegenerative diseases [113,114,115]. Since the brain is more susceptible to oxidants than other organs, oxidative damage frequently coexists with neurodegenerative disorders. In PD, increased monoamine oxidase B (MAO-B) levels and impaired mitochondrial activity are linked to elevations in ROS generation [116,117,118] and cellular damage [118,119], which are responsible for neurodegeneration and neuroinflammation [118,120]. Altered glutathione levels in the dopaminergic neurons of transgenic mice provide more evidence for the hypothesis that oxidative stress drives neuronal loss in PD [121].

In LPS-stimulated microglia, TAN exerts an antioxidant impact by reducing ROS production while boosting heme oxygenase-1 (HO-1) expression [121] via the nuclear factor erythroid 2-related factor 2 (Nrf2) signaling pathway, which is one of the therapeutic targets of PD [122]. It has recently been determined that moderate mitochondrial depolarization, which prevents neurotoxic mitochondrial calcium overload during neural insults, represents a unique neuroprotective mechanism. Due to its inhibitory actions in (H_2_O_2_)-induced mitochondrial calcium ion (Ca^2+^) accumulation, TAN significantly boosts HT-22 neuron survival of H_2_O_2_-induced cell death in a dose-dependent manner, thus preventing neuronal apoptosis [123]. Furthermore, TAN was shown to substantially inhibit ultraviolet B (UVB)-induced ROS production in JB6 P+ cells; this ability could be used to restrict endogenous ROS production [124].

In the kidney tissue, TAN significantly decreased lipid peroxides, and inflammatory cytokines, further improving the levels of both enzymatic and nonenzymatic antioxidants [125,126]. TAN effectively normalized the antioxidant enzymes SOD, CAT, GPx, and GR in diabetic rats [127].

### 4.2. Anti-Inflammatory Effect of TAN

Inflammation is a typical physiologic reaction to tissue damage, microbial pathogen infection, and chemical irritation. However, inflammatory mediators, including free radicals, cytokines, and chemokines, will infiltrate the body and be produced in excessive amounts, damaging cellular and tissue functions. PGE2 and COX-2 are created by the inducible enzyme COX, which also plays a crucial role in the creation of PGE2 in inflammatory areas and the unregulated NO produced by the iNOS. The CNS macrophages, known as microglial cells, maintain the nervous system’s balance by removing damaged neurons and preventing the spread of infection. This macrophage population produces cytokines that cause inflammation, creating ROS as the first line of defense against microorganisms [128]. However, a pro-inflammatory and pro-oxidant condition sustained over time by chronic microglia activation is harmful because it may speed up the neurodegenerative process via neuroinflammation, as occurs in the case of AD and PD [129,130].

Chemokine fractalkine (CX3CL1), produced mainly by neurons, has recently been suggested as a potential biomarker for PD [131]. The interaction between neurons and glial cells is mediated by the G-protein-coupled receptor CX3CL1, a regulator of microglial activity [131]. Additionally, older adults with PD have been reported to have peripheral inflammation, which is indicated by an increase in the blood levels of IL-8 and MIP-1 and reduction in the levels of IL-9 and MIP-1 [132].

**Figure 5:**
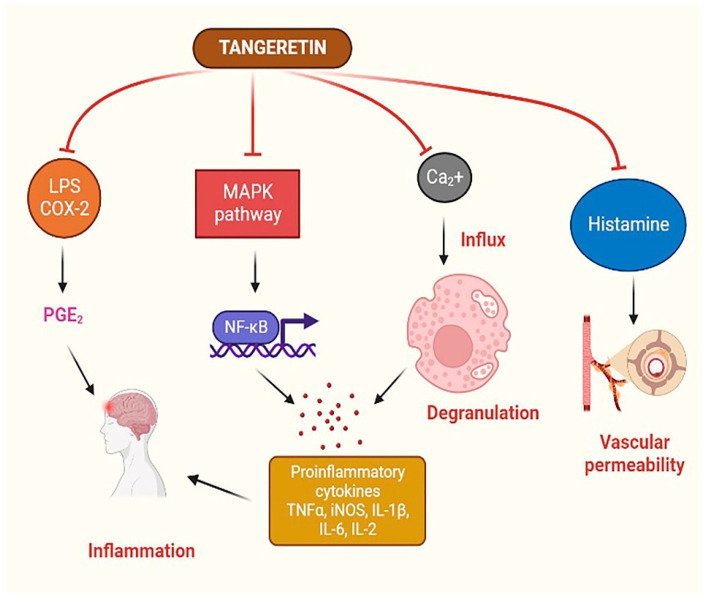
TAN’s anti-inflammatory mechanisms. TAN reduces the production of proinflammatory cytokines by controlling certain essential enzymes involved in the MAPK pathway and cell degranulation. TAN also interferes with the formation of PGE2 generated by LPS and COX-2 and reduces vascular permeability caused by histamine to prevent allergies. TAN: tangeretin; MAPK: mitogen-activated protein kinases; PGE2: prostaglandin E2; COX: cyclooxygenase; LPS: lipopolysaccharide; IL: interleukin; TNF-α: tumor necrosis factor-alpha; iNOS: inducible nitric oxide synthase; NF-κB: nuclear factor-kappa B.

Evidence suggests that the key signaling pathway for TAN’s neuroprotective benefits might be via its anti-inflammatory properties. TAN showed a solid ability to suppress neuroinflammation caused by microglial activation stimulated by lipopolysaccharide [97]. These results indicate that TAN inhibits the production of COX-2 and iNOS at the transcriptional and protein levels in microglial cells. The production of proinflammatory substances, including NO, IL-1, TNF-α, and IL-6, was also inhibited in a dose-dependent fashion. Thus, IκB kinase and MAPKs may function separately or jointly to control TAN’s inhibition of proinflammatory mediators [97]. MMPs are vital for many physiological tasks, including proliferation, differentiation, cell motility, apoptosis, and host defense. Blood–brain barrier (BBB) collapse, neuronal cell death, and peripheral immune cell infiltration in neuropathological illnesses, including AD, PD, and MS, are all unfavorable outcomes of abnormal MMP expression. MMPs influence the development of neuroinflammatory diseases by modulating TNF-α activation. TAN reduces MMPs, including MMP-3 and MMP-8, in LPS-stimulated microglia, which aids its anti-inflammatory effects. A schematic diagram of the possible anti-inflammatory mechanism of TAN is presented in Figure 5.

### 4.3. Diabetes-Mediated Neurodegeneration and TAN

The pathophysiology of Diabetes Mellitus (DM) and neurodegeneration is influenced by environmental and genetic variations [133]. Some DM animal models have comparable clinical traits to PD and AD animal models [133,134]. DM and neurodegeneration have the characteristics of impaired glucose metabolism, insulin resistance, and mitochondrial dysfunction, which manifests before clinical diagnosis of neurodegenerative illness. Other causes of the pathophysiology of both disorders include microbial dysbiosis, the aggregation of misfolded proteins, persistent inflammation, and oxidative stress [135]. Dopaminergic neurons and insulin receptors coexist in the substantia nigra, supporting the hypothesis that DM and PD are directly related. The reduced insulin signaling in the basal ganglia correlates with dopamine depletion in the striatum [136]. DM is also associated with a significant decline in cognitive function and the elevated susceptibility to dementia seen in AD. Several common pathways, including apoptosis, neuron aging, lipid peroxidation, tau phosphorylation, and brain shrinkage, are all adversely affected in DM-mediated AD. One possible strategy to treat AD is to repurpose anti-diabetic agents as beneficial therapeutics that may avert or lower the risk of cognitive decline and neurodegeneration [137,138].

Experimental studies in diabetic rats showed a decrease in plasma glucose and glycosylated hemoglobin (HbA1c) levels, although the hemoglobin and insulin levels drastically increased because of TAN. The liver’s major enzyme processes for glycolysis, glycogenolysis, the pentose phosphate pathway, and glycogenesis were nearly fully recovered [139]. TAN decreased the capacity of cells to secrete insulin, reduced the amounts of ROS and the messenger RNA in insulin 1 and 2, and enhanced the expression of SOD, CAT, and GPX activities to prevent streptozotocin-induced apoptosis. TAN’s protective effects resulted from its blocking the NF-κB signaling pathway [140].

**Figure 6:**
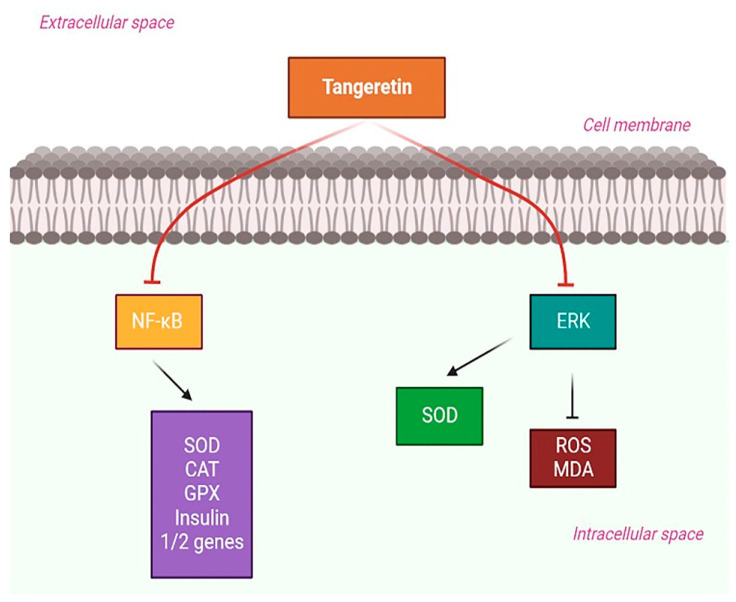
Possible signaling pathways showing the effect of TAN against Diabetes Miletus-mediated neuroinflammation. TAN: tangeretin; MDA: malondialdehyde; NF-κB: nuclear factor kappa B; CAT: catalase; GPX: glutathione peroxidase; ERK: extracellular-signal-regulated kinase.

In another study, Chen et al., investigated how TAN inhibits human renal mesangial cells (MC) extracellular matrix production in response to high glucose levels. TAN substantially boosted SOD activity while decreasing MC growth, ROS, and MDA levels. TAN partially inhibited the ERK signaling pathway to provide these protective effects [141]. Based on these reports, we can conclude that TAN can aid in countering DM-mediated neuroinflammatory responses and can provide therapeutic benefits in the management of neurodegenerative disorders. The possible role of TAN in DM-mediated neuroinflammation is depicted in Figure 6.

### 4.4. Peroxisome Proliferator Receptor-Gamma (PPAR-γ) Agonistic Effects of TAN

The nuclear receptor superfamily, which includes PPAR-γ, controls mitochondrial activity and modifies lipid and glucose metabolism. PPAR-γ agonists, such as pioglitazone, lower inflammation by preventing the production of IL-6 and TNF-α [142]. In the brains of MPTP-treated monkeys, pioglitazone reduced inflammatory responses while maintaining dopaminergic nigrostriatal function [143]. Additionally, pioglitazone treatment reduced the glial activation and arrested the loss of dopaminergic neurons in the substantia nigra (SN) of mice treated with MPTP [144,145]. In the SN of mice treated with MPTP, rosiglitazone, another PPAR-γ agonist, reversed the loss of dopaminergic neurons [146]. These findings suggest the use of PPAR agonists as potential anti-inflammatory treatments to slow the course of neurodegeneration. TAN suppressed UVB-induced COX-2 expression and PGE2 production in human epidermal keratinocytes (HaCaT) cells through PPAR-γ activation [147]. Further, Kim et al., Li et al., and Kurowska et al. have also reported on the PPAR-agonistic activities of TAN in their studies; thus, these effects of TAN can halt the progression of PD [148,149,150].

## 5. Limitations and Future Perspectives

One of the main issues with flavonoids is the length of time required for epidemiological investigations due to the extensive exposure times, data collection, and analyses of the presence and absence of flavonoids that are required. Due to their very low yield (mg–gm per kg of plant weight) and the fact that they must be continuously extracted from *Citrus* plants to avoid extinction, TAN’s production cost is another significant concern. Such issues might be resolved by a targeted study on the production of flavonoids by microbes or any other natural sources. The purification procedure presents another significant challenge because flavonoids generally tend to form tight complexes with other secondary metabolites, vitamins, minerals, and fibers, making it challenging to separate them.

**Figure 7:**
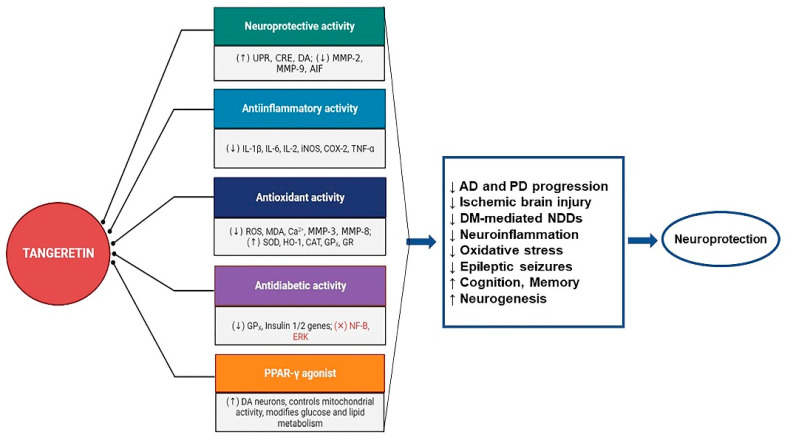
TAN’s potential as a candidate for treatments of neuroinflammation-mediated neurodegenerative disorders. AD: Alzheimer’s disease; PD: Parkinson’s disease; DM: Diabetes Miletus; NDDs: neurodegenerative disorders; UPR: unfolded protein response; CRE: cAMP response element; DA: dopamine; MMP: matrix metalloproteinases; AIF: apoptosis-inducing factor; IL: interleukin; iNOS: inducible nitric oxide synthase; COX: cyclooxygenase; TNF-α: tumor necrosis factor-alpha; ROS: reactive oxygen species; MDA: malondialdehyde; SOD: superoxide dismutase; HO: heme oxygenase; CAT: catalase; GPx: glutathione peroxidase; GR: glutathione reductase; NF-κB: nuclear factor-kappa B; ERK: extracellular-signal-regulated kinase; ↑: increase; ↓: decrease; ×: inhibited.

In order to improve the purity of the separated flavonoids and allow for shorter extraction procedures with a lower cost, it is also necessary to create an effective purification method. TAN’s low water solubility, as a result of its lipophilic makeup, is another serious issue. To boost its bioavailability in the studied animal systems, future research should concentrate on various targeted delivery methods, including the novel nano-emulsion-based delivery systems. In addition, a detailed, pharmacokinetic, dosage regimen should be established, as well as research on the safety issues and toxicity profile, and pre-clinical research in various other neuroinflammatory animal models, to better understand the intrinsic mechanisms of TAN. To the best of our knowledge and understanding, no published clinical data are yet available on the impact of the chronic long-term intake of TAN and its neuroprotective benefits. To close these gaps, a systematic research plan of action, extending pre-clinical observations into clinical settings, is necessary to demonstrate TAN’s neuroprotective potential. An illustrative diagram of the possible overall benefits of TAN as a potential agent against neuroinflammation-mediated neurodegenerative disorders is presented in Figure 7.

## 6. Conclusions

Based on the available literature and reported studies, TAN, one of the active flavonoids present in *Citrus* species of plants, has the potential to prevent neuronal death in various neurodegenerative diseases. Due to TAN’s excellent physiochemical and pharmacokinetic profile, TAN is a promising candidate and can be used as an auxiliary therapy to prevent or delay the progression of neuroinflammation-mediated neurodegenerative disorders.

## Figures and Tables

**Table 2:** Physicochemical properties of TAN.

Properties	Values
Physical Description	Solid
HBDC	0
HBAC	7
BP	566 °C @ 760 mm Hg
MP	154 °C
Solubility	8.70 mg/L @ 25 °C
LogP	1.78

Abbreviations: HBDC: hydrogen bond donor count; HBAC: hydrogen bond acceptor count; BP: boiling point; MP: melting point.

**Table 3:** Summary of established neuroprotective effects of TAN in experimental models of neurodegeneration.

No.	Model	Experimental Design/Dose	Parameters Tested	Mechanism	Conclusion	Ref.
1	6-OHDA-induced PD rat model	20 mg/kg/day for 4 days; p.o.	TH^+^ cells and striatal dopamine content	Reduced TH^+^ cells; increased striatal dopamine content	Neuroprotective agent	[21]
2	MPTP-induced rat PD model	50, 100 or 200 mg/kg body weight for 20 days	Rotarod, working memory, object recognition, inflammatory mediators, cytokines	Enhanced memory and locomotion; decreased COX-2, iNOS, IL-1β, IL-6, and IL-2	Neuroinflammation and dementia associated with PD	[48]
3	Transgenic Drosophila PD model	5, 10 and 20 µM in diet for 24 days	Climbing ability, dopamine levels, antioxidant enzymes	Enhanced climbing ability and dopamine content; decreased oxidative stress	Enhanced behavioral pattern and antioxidant	[100]
4	Pilocarpine-induced mice	50, 100, or 200 mg/kg for 10 days	Neuronal apoptosis and seizure severity	Regulation of PI3K/Akt signalling; decreased seizure-induced MMP-2, MMP-9, and AIF	Recovered PD-associated epileptic seizures	[102]
5	APPswe/PSEN1dE9 transgenic AD mice model	100 mg/kg body weight/day	Cognitive functions, Aβ aggregation	Inhibited β-secretase both in vitro and in vivo	Anti-dementia effect	[22]
6	Aβ-induced rat primary neurons	25 µM	Oxidative damage, Aβ aggregation	Reduced free radical damage and suppressed Aβ neurotoxicity	Neuroprotective effect	[103]
7	HepG2 cells in vitro and ischemic-reperfusion rat model	100 μg/mL for in vitro and 200 mg/kg in vivo	Apoptosis and cell viability	Inhibited apoptosis, and reduced brain injury	Neuroprotection and ischemic stroke protection	[18]
8	OGD insult in HBMEC cells	2.5, 5 and 10 µM	Cell viability, ROS levels, inflammatory pathways	Reduced ROS levels; ameliorated apoptosis; regulated JNK signaling	Protects brain injury and related neurogenerative diseases	[106]
9	Global cerebral ischemia in rats	5,10, and 20 mg/kg, oral	Cognition and memory, AchE, Ach levels, ROS levels, inflammation markers	Increased memory and cognition; attenuated AchE and Ach activities; inhibited IL-6 and TNF-α, mitigating apoptosis	Neuroprotection, and antineuroinflammation	[24]
10	In vivo MCAO/R mice model and OGD/R injury in hippocampal HT22 cell in vitro	5, 10 and 20 µM in vitro and 10 µM in vivo	Cell viability, neuronal pyroptosis	Attenuated pyroptosis and regulated Nrf-2 signaling	Neuroprotective effects	[107]
11	LM mice model	5, 10 and 15 mg/kg	Cognitive functions, novel object recognition, inflammatory mediators	Recovered cognitio; decreased ERK ½, TNFα; and IL-1β expression; modulated RORα/γ target genes	Cognitive deficiency and related diseases	[23]
12	Potassium dichromate -induced brain injury in rats	50 mg/kg; orally, for 14 days	Behavioral indices, ROS markers, inflammatory markers	Reduced ROS levels; inhibited TNF-α and IL-6; regulated Nrf2 signaling pathway	Neuroprotective effect, anti-neuroinflammation, antioxidant	[108]

Abbreviations: 6-OHDA: 6-Hydroxydopamine, MPTP: 1-methyl-4-phenyl-1,2,3,6-tetrahydropyridine; MMP: matrix metalloproteinases; AIF: apoptosis-inducing factor; Aβ: amyloid beta; HepG2: human hepatocellular carcinoma cells; HBMECs: human brain microvascular endothelial cells; OGD: oxygen–glucose deprivation; AchE: acetylcholine esterase; Ach: acetylcholine; MCAO/R: middle cerebral artery occlusion/reperfusion; OGD/R: oxygen–glucose deprivation and reoxygenation; LPS: lipopolysaccharide; LM: LPS plus midazolam; PD: Parkinson’s disease; AD: Alzheimer’s disease; ROS: reactive oxygen species; TNF-α: tumor necrosis factor-alpha; IL: interleukin; Nrf-2: nuclear factor erythroid 2-related factor 2; TAN: tangeretin; TH^+^: tyrosine hydroxylase positive; COX- cyclooxygenase; iNOS: inducible nitric oxide synthase; PI3K: phosphatidylinositol-3-kinase; AKT: protein kinase B; JNK: c-Jun N-terminal kinase; ERK ½: extracellular-signal-regulated kinase ½; RORα/γ: retinoic-acid-receptor-related orphan receptors α and **γ**.

## Data Availability

Not applicable.
